# 2-Formyl-6-meth­oxy­phenyl cinnamate

**DOI:** 10.1107/S160053681202747X

**Published:** 2012-06-23

**Authors:** N. Manikandan, S. Murugavel, D. Kannan, M. Bakthadoss

**Affiliations:** a Department of Physics, Bharathidasan Engineering College, Nattrampalli, Vellore 635 854, India; b Department of Physics, Thanthai Periyar Government Institute of Technology, Vellore 632 002, India; c Department of Organic Chemistry, University of Madras, Maraimalai Campus, Chennai 600 025, India

## Abstract

In the title compound, C_17_H_14_O_4_, the C=C bond adopts an *E* conformation and the dihedral angle between the benzene rings is 73.9 (1)°. The crystal packing features C—H⋯O hydrogen bonds, which generate *C*(4) chains propagating along the *b*-axis direction. Weak aromatic π–π stacking inter­actions [centroid–centroid distance = 3.703 (1) Å] are also observed.

## Related literature

For the biological properties of cinnamate derivatives, see: Sharma (2011 ▶). For related structures, see: Kaitner & Stilinović (2007 ▶); Anuradha *et al.* (2012 ▶).
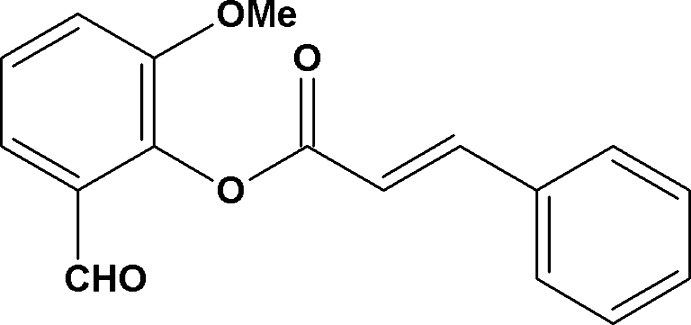


## Experimental

### 

#### Crystal data

C_17_H_14_O_4_
*M* *_r_* = 282.28
Orthorhombic, 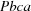

*a* = 10.7908 (7) Å
*b* = 10.4672 (5) Å
*c* = 25.8714 (17) Å
*V* = 2922.2 (3) Å^3^
*Z* = 8
Mo *K*α radiation
μ = 0.09 mm^−1^
*T* = 293 K
0.23 × 0.21 × 0.15 mm

#### Data collection

Bruker APEXII CCD diffractometer
Absorption correction: multi-scan (*SADABS*; Sheldrick, 1996 ▶) *T*_min_ = 0.910, *T*_max_ = 0.941
14344 measured reflections
2665 independent reflections
1909 reflections with *I* > 2σ(*I*)
*R*_int_ = 0.026

#### Refinement

*R*[*F* ^2^ > 2σ(*F* ^2^)] = 0.037
*wR*(*F* ^2^) = 0.106
*S* = 1.01
2665 reflections
192 parameters
H-atom parameters constrained
Δρ_max_ = 0.15 e Å^−3^
Δρ_min_ = −0.12 e Å^−3^

### 

Data collection: *APEX2* (Bruker, 2004 ▶); cell refinement: *APEX2* and *SAINT* (Bruker, 2004 ▶); data reduction: *SAINT* and *XPREP* (Bruker, 2004 ▶); program(s) used to solve structure: *SHELXS97* (Sheldrick, 2008 ▶); program(s) used to refine structure: *SHELXL97* (Sheldrick, 2008 ▶); molecular graphics: *ORTEP-3* (Farrugia (1997 ▶); software used to prepare material for publication: *SHELXL97* and *PLATON* (Spek, 2009 ▶).

## Supplementary Material

Crystal structure: contains datablock(s) global, I. DOI: 10.1107/S160053681202747X/hb6858sup1.cif

Structure factors: contains datablock(s) I. DOI: 10.1107/S160053681202747X/hb6858Isup2.hkl

Supplementary material file. DOI: 10.1107/S160053681202747X/hb6858Isup3.cml

Additional supplementary materials:  crystallographic information; 3D view; checkCIF report

## Figures and Tables

**Table 1:** Hydrogen-bond geometry (Å, °)

*D*—H⋯*A*	*D*—H	H⋯*A*	*D*⋯*A*	*D*—H⋯*A*
C9—H9⋯O3^i^	0.93	2.50	3.415 (2)	168

Symmetry code: (i) 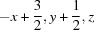.

**Fig. 1.**
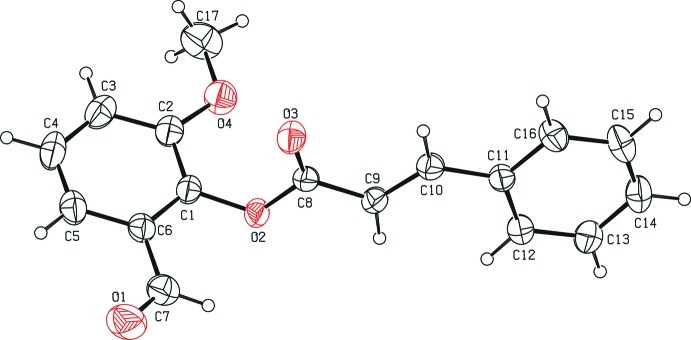
Molecular structure of the title compound showing displacement ellipsoids at the 30% probability level.

**Fig. 2.**
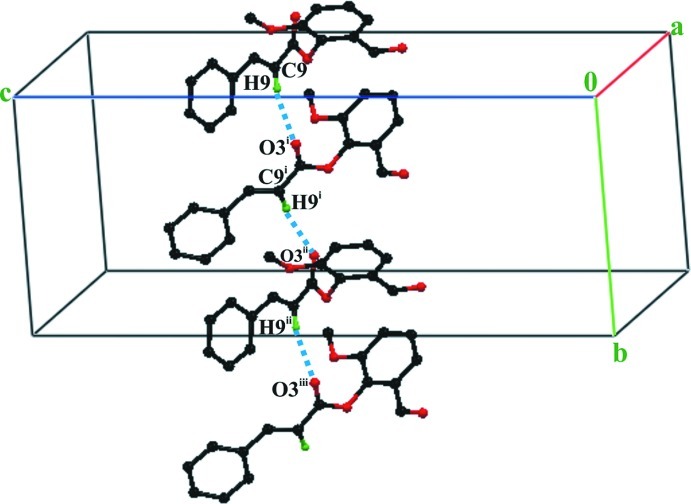
Part of the crystal structure of (I) showing intermolecular C—H···O hydrogen bonds (dotted lines), forming C(4) chains along the b axis. For clarity H atoms involved in the hydrogen bonds are shown. [Symmetry codes:(i)3/2 - x, 1/2 + y, z; (ii)x, 1 + y, z; (iii)3/2 - x, 3/2 + y, z].

**Fig. 3.**
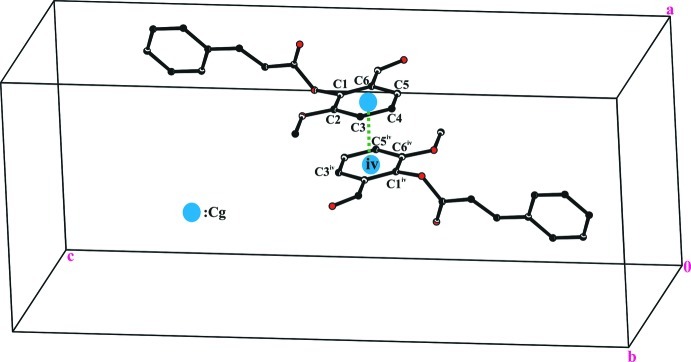
A view of the π—π interactions (dotted lines) in the crystal structure of the title compound. Cg denotes centroid of the C1–C6 benzene ring. [Symmetry code: (iv)1 - x, -y, 1 - z].

## supplementary crystallographic information

### Comment

Cinnamic acid and its derivatives including esters and carboxylic functional derivatives are used as important components in flavours, perfumes, synthetic indigo and pharmaceuticals. Cinnamate can act as optical filters or deactivate substrate molecules that have been excited by light for the protection polymers and organic substances. They are used as cosmetic grades and as sunscreen agents to reduce skin damage by blocking UV—A, B (Sharma, 2011). As part of our studies in this area, the crystal structure determination of the title compound was carried out and the results are presented here.

The C9=C10 double bond of the title compound (I) exists in an *E*-configuration (Fig. 1). The dihedral angle between the two benzene rings is 73.9 (1)°. The geometric parameters of the title molecule agrees well with those reported for similar structures (Kaitner *et al.*, 2007, Anuradha *et al.*, 2012).

The crystal packing features C—H···O hydrogen bonds. Atom C9 at *x, y, z* donates one proton to atom O3 at *3/2 - x, 1/2 + y, z*, forming C(4) chains along the *b* axis (Fig. 2). The crystal packing (Fig. 3) also features π—π interactions with a *Cg*—*Cg*^iv^ seperation of 3.703 (1) Å.[Fig. 3; *Cg* is the centroid of the C1–C6 benzene ring, symmetry code as in Fig. 3].

### Experimental

To a stirred solution of 2-hydroxy-3-methoxybenzaldehyde (1 mmol, 0.154 g) and potassium carbonate (1.5 mmol, 0.207 g) was stirred for 15 minutes in acetonitrile as solvent at room temperature. To this solution, (2*E*)-3-phenylprop-2-enoylchloride (1 mmol, 0.167 g) was added till the addition is complete. After the completion of the reaction as indicated by TLC, acetonitrile solvent was evaporated. Ethylacetate (15 ml) and water (15 ml) were added to the crude mass. The organic layer was dried over anhydrous sodium sulfate. Removal of solvent led to the crude product. The pure title compound was obtained as a colorless solid (0.250 g, 89% yield). Recrystallization was carried out using ethylacetate as solvent to yield colourless blocks.

### Refinement

H atoms were positioned geometrically, with C–H = 0.93–0.96 Å and constrained to ride on their parent atoms, with *U*_iso_(H) =1.5*U*_eq_ for methyl H atoms and 1.2*U*_eq_(C) for other H atoms.

### Crystal data

**Table:**

C_17_H_14_O_4_	*F*(000) = 1184
*M**_r_* = 282.28	*D*_x_ = 1.283 Mg m^−^^3^
Orthorhombic, *P**b**c**a*	Mo *K*α radiation, λ = 0.71073 Å
Hall symbol: -P 2ac 2ab	Cell parameters from 2879 reflections
*a* = 10.7908 (7) Å	θ = 1.0–1.0°
*b* = 10.4672 (5) Å	µ = 0.09 mm^−^^1^
*c* = 25.8714 (17) Å	*T* = 293 K
*V* = 2922.2 (3) Å^3^	Block, colourless
*Z* = 8	0.23 × 0.21 × 0.15 mm

### Data collection

**Table:**

Bruker APEXII CCD diffractometer	2665 independent reflections
Radiation source: fine-focus sealed tube	1909 reflections with *I* > 2σ(*I*)
Graphite monochromator	*R*_int_ = 0.026
Detector resolution: 10.0 pixels mm^-1^	θ_max_ = 26.0°, θ_min_ = 2.5°
ω scans	*h* = −13→13
Absorption correction: multi-scan (*SADABS*; Sheldrick, 1996)	*k* = −9→11
*T*_min_ = 0.910, *T*_max_ = 0.941	*l* = −31→31
14344 measured reflections	

### Refinement

**Table:**

Refinement on *F*^2^	Secondary atom site location: difference Fourier map
Least-squares matrix: full	Hydrogen site location: inferred from neighbouring sites
*R*[*F*^2^ > 2σ(*F*^2^)] = 0.037	H-atom parameters constrained
*wR*(*F*^2^) = 0.106	*w* = 1/[σ^2^(*F*_o_^2^) + (0.0446*P*)^2^ + 0.7231*P*] where *P* = (*F*_o_^2^ + 2*F*_c_^2^)/3
*S* = 1.01	(Δ/σ)_max_ = 0.001
2665 reflections	Δρ_max_ = 0.15 e Å^−^^3^
192 parameters	Δρ_min_ = −0.12 e Å^−^^3^
0 restraints	Extinction correction: *SHELXL97* (Sheldrick, 2008), Fc^*^=kFc[1+0.001xFc^2^λ^3^/sin(2θ)]^-1/4^
Primary atom site location: structure-invariant direct methods	Extinction coefficient: 0.0020 (5)

### Special details

**Table:**

Geometry. All e.s.d.'s (except the e.s.d. in the dihedral angle between two l.s. planes) are estimated using the full covariance matrix. The cell e.s.d.'s are taken into account individually in the estimation of e.s.d.'s in distances, angles and torsion angles; correlations between e.s.d.'s in cell parameters are only used when they are defined by crystal symmetry. An approximate (isotropic) treatment of cell e.s.d.'s is used for estimating e.s.d.'s involving l.s. planes.
Refinement. Refinement of *F*^2^ against ALL reflections. The weighted *R*-factor *wR* and goodness of fit *S* are based on *F*^2^, conventional *R*-factors *R* are based on *F*, with *F* set to zero for negative *F*^2^. The threshold expression of *F*^2^ > σ(*F*^2^) is used only for calculating *R*-factors(gt) *etc*. and is not relevant to the choice of reflections for refinement. *R*-factors based on *F*^2^ are statistically about twice as large as those based on *F*, and *R*- factors based on ALL data will be even larger.

### Fractional atomic coordinates and isotropic or equivalent isotropic displacement parameters (Å^2^)

**Table:**

	*x*	*y*	*z*	*U*_iso_*/*U*_eq_	
C1	0.62871 (15)	−0.06915 (15)	0.54858 (6)	0.0487 (4)	
C2	0.54050 (16)	−0.15242 (16)	0.56771 (7)	0.0552 (4)	
C3	0.48566 (18)	−0.23727 (18)	0.53357 (8)	0.0686 (5)	
H3	0.4252	−0.2935	0.5453	0.082*	
C4	0.5205 (2)	−0.2388 (2)	0.48191 (8)	0.0732 (6)	
H4	0.4827	−0.2959	0.4593	0.088*	
C5	0.60927 (19)	−0.15776 (18)	0.46393 (7)	0.0649 (5)	
H5	0.6326	−0.1603	0.4293	0.078*	
C6	0.66465 (16)	−0.07149 (16)	0.49726 (7)	0.0532 (4)	
C7	0.7586 (2)	0.0175 (2)	0.47767 (8)	0.0702 (5)	
H7	0.7865	0.0813	0.4998	0.084*	
C8	0.76334 (15)	−0.01222 (16)	0.61648 (6)	0.0459 (4)	
C9	0.78497 (15)	0.08922 (16)	0.65399 (6)	0.0491 (4)	
H9	0.7521	0.1700	0.6480	0.059*	
C10	0.85036 (15)	0.06857 (17)	0.69633 (6)	0.0509 (4)	
H10	0.8863	−0.0119	0.6993	0.061*	
C11	0.87318 (14)	0.15626 (16)	0.73902 (6)	0.0484 (4)	
C12	0.82650 (17)	0.28000 (17)	0.73974 (6)	0.0595 (5)	
H12	0.7812	0.3101	0.7117	0.071*	
C13	0.84677 (18)	0.35820 (19)	0.78160 (7)	0.0663 (5)	
H13	0.8149	0.4407	0.7816	0.080*	
C14	0.91335 (17)	0.3161 (2)	0.82338 (7)	0.0667 (5)	
H14	0.9258	0.3694	0.8517	0.080*	
C15	0.96137 (18)	0.1953 (2)	0.82313 (7)	0.0703 (6)	
H15	1.0074	0.1665	0.8512	0.084*	
C16	0.94166 (17)	0.11608 (19)	0.78136 (6)	0.0619 (5)	
H16	0.9749	0.0341	0.7816	0.074*	
C17	0.41618 (19)	−0.2177 (2)	0.63873 (9)	0.0923 (7)	
H17A	0.3401	−0.1947	0.6219	0.138*	
H17B	0.4085	−0.2032	0.6752	0.138*	
H17C	0.4336	−0.3063	0.6325	0.138*	
O1	0.80202 (17)	0.01350 (16)	0.43495 (6)	0.0987 (5)	
O2	0.67581 (11)	0.02494 (10)	0.58150 (4)	0.0542 (3)	
O3	0.81108 (12)	−0.11529 (12)	0.61497 (5)	0.0630 (4)	
O4	0.51428 (13)	−0.14196 (13)	0.61881 (5)	0.0731 (4)	

### Atomic displacement parameters (Å^2^)

**Table:**

	*U*^11^	*U*^22^	*U*^33^	*U*^12^	*U*^13^	*U*^23^
C1	0.0519 (10)	0.0386 (10)	0.0557 (9)	0.0070 (7)	−0.0156 (8)	−0.0053 (7)
C2	0.0530 (10)	0.0508 (11)	0.0617 (10)	0.0072 (8)	−0.0079 (8)	−0.0057 (8)
C3	0.0582 (11)	0.0561 (12)	0.0914 (15)	−0.0041 (9)	−0.0122 (10)	−0.0078 (10)
C4	0.0754 (13)	0.0635 (14)	0.0806 (14)	0.0058 (11)	−0.0266 (11)	−0.0246 (11)
C5	0.0766 (13)	0.0622 (13)	0.0560 (10)	0.0125 (11)	−0.0150 (10)	−0.0123 (9)
C6	0.0599 (11)	0.0471 (11)	0.0527 (9)	0.0099 (8)	−0.0109 (8)	−0.0010 (8)
C7	0.0815 (14)	0.0650 (14)	0.0641 (11)	0.0035 (11)	−0.0039 (11)	0.0060 (10)
C8	0.0482 (9)	0.0427 (11)	0.0468 (8)	−0.0014 (8)	−0.0038 (7)	0.0034 (7)
C9	0.0550 (10)	0.0404 (10)	0.0520 (9)	−0.0011 (8)	−0.0064 (8)	−0.0009 (7)
C10	0.0528 (10)	0.0467 (11)	0.0531 (9)	0.0010 (8)	−0.0034 (8)	0.0018 (7)
C11	0.0483 (9)	0.0520 (11)	0.0449 (8)	−0.0040 (8)	−0.0014 (7)	0.0005 (7)
C12	0.0710 (12)	0.0564 (12)	0.0510 (10)	−0.0017 (9)	−0.0106 (9)	−0.0022 (8)
C13	0.0793 (13)	0.0588 (12)	0.0607 (11)	−0.0032 (10)	−0.0011 (10)	−0.0107 (9)
C14	0.0587 (11)	0.0911 (16)	0.0502 (10)	−0.0126 (11)	0.0015 (9)	−0.0172 (10)
C15	0.0601 (11)	0.1040 (17)	0.0469 (9)	0.0032 (11)	−0.0090 (8)	−0.0033 (10)
C16	0.0625 (11)	0.0700 (13)	0.0532 (10)	0.0090 (9)	−0.0073 (9)	0.0018 (9)
C17	0.0650 (13)	0.118 (2)	0.0936 (16)	−0.0043 (13)	0.0096 (12)	0.0217 (14)
O1	0.1247 (14)	0.0973 (12)	0.0741 (10)	−0.0031 (10)	0.0230 (9)	0.0072 (8)
O2	0.0664 (7)	0.0407 (7)	0.0556 (6)	0.0052 (5)	−0.0191 (6)	−0.0055 (5)
O3	0.0680 (8)	0.0495 (8)	0.0717 (8)	0.0140 (6)	−0.0187 (6)	−0.0094 (6)
O4	0.0713 (9)	0.0803 (10)	0.0678 (8)	−0.0064 (7)	0.0058 (7)	−0.0038 (7)

### Geometric parameters (Å, º)

**Table:**

C1—C2	1.382 (2)	C9—H9	0.9300
C1—C6	1.383 (2)	C10—C11	1.457 (2)
C1—O2	1.3978 (19)	C10—H10	0.9300
C2—O4	1.356 (2)	C11—C16	1.387 (2)
C2—C3	1.385 (2)	C11—C12	1.390 (2)
C3—C4	1.389 (3)	C12—C13	1.375 (2)
C3—H3	0.9300	C12—H12	0.9300
C4—C5	1.361 (3)	C13—C14	1.371 (3)
C4—H4	0.9300	C13—H13	0.9300
C5—C6	1.384 (2)	C14—C15	1.366 (3)
C5—H5	0.9300	C14—H14	0.9300
C6—C7	1.468 (3)	C15—C16	1.379 (3)
C7—O1	1.201 (2)	C15—H15	0.9300
C7—H7	0.9300	C16—H16	0.9300
C8—O3	1.1961 (19)	C17—O4	1.419 (2)
C8—O2	1.3646 (18)	C17—H17A	0.9600
C8—C9	1.457 (2)	C17—H17B	0.9600
C9—C10	1.321 (2)	C17—H17C	0.9600
			
C2—C1—C6	121.71 (15)	C9—C10—H10	116.0
C2—C1—O2	118.45 (15)	C11—C10—H10	116.0
C6—C1—O2	119.70 (15)	C16—C11—C12	117.72 (15)
O4—C2—C1	116.23 (15)	C16—C11—C10	119.85 (16)
O4—C2—C3	125.74 (18)	C12—C11—C10	122.41 (15)
C1—C2—C3	118.03 (17)	C13—C12—C11	120.51 (17)
C2—C3—C4	120.37 (19)	C13—C12—H12	119.7
C2—C3—H3	119.8	C11—C12—H12	119.7
C4—C3—H3	119.8	C14—C13—C12	120.87 (19)
C5—C4—C3	120.80 (18)	C14—C13—H13	119.6
C5—C4—H4	119.6	C12—C13—H13	119.6
C3—C4—H4	119.6	C15—C14—C13	119.51 (18)
C4—C5—C6	119.83 (18)	C15—C14—H14	120.2
C4—C5—H5	120.1	C13—C14—H14	120.2
C6—C5—H5	120.1	C14—C15—C16	120.12 (18)
C1—C6—C5	119.24 (18)	C14—C15—H15	119.9
C1—C6—C7	120.93 (16)	C16—C15—H15	119.9
C5—C6—C7	119.82 (17)	C15—C16—C11	121.26 (18)
O1—C7—C6	124.4 (2)	C15—C16—H16	119.4
O1—C7—H7	117.8	C11—C16—H16	119.4
C6—C7—H7	117.8	O4—C17—H17A	109.5
O3—C8—O2	122.24 (14)	O4—C17—H17B	109.5
O3—C8—C9	127.58 (15)	H17A—C17—H17B	109.5
O2—C8—C9	110.17 (14)	O4—C17—H17C	109.5
C10—C9—C8	121.24 (16)	H17A—C17—H17C	109.5
C10—C9—H9	119.4	H17B—C17—H17C	109.5
C8—C9—H9	119.4	C8—O2—C1	117.06 (12)
C9—C10—C11	128.01 (16)	C2—O4—C17	117.68 (16)
			
C6—C1—C2—O4	179.15 (15)	C8—C9—C10—C11	−175.41 (15)
O2—C1—C2—O4	−5.3 (2)	C9—C10—C11—C16	178.04 (17)
C6—C1—C2—C3	−1.7 (2)	C9—C10—C11—C12	−0.6 (3)
O2—C1—C2—C3	173.89 (14)	C16—C11—C12—C13	−0.9 (3)
O4—C2—C3—C4	179.98 (17)	C10—C11—C12—C13	177.80 (17)
C1—C2—C3—C4	0.9 (3)	C11—C12—C13—C14	0.1 (3)
C2—C3—C4—C5	0.3 (3)	C12—C13—C14—C15	0.8 (3)
C3—C4—C5—C6	−0.8 (3)	C13—C14—C15—C16	−0.7 (3)
C2—C1—C6—C5	1.2 (2)	C14—C15—C16—C11	−0.1 (3)
O2—C1—C6—C5	−174.30 (14)	C12—C11—C16—C15	0.9 (3)
C2—C1—C6—C7	−179.88 (16)	C10—C11—C16—C15	−177.80 (17)
O2—C1—C6—C7	4.6 (2)	O3—C8—O2—C1	10.1 (2)
C4—C5—C6—C1	0.0 (3)	C9—C8—O2—C1	−169.00 (14)
C4—C5—C6—C7	−178.85 (17)	C2—C1—O2—C8	78.41 (18)
C1—C6—C7—O1	173.14 (19)	C6—C1—O2—C8	−105.90 (17)
C5—C6—C7—O1	−8.0 (3)	C1—C2—O4—C17	174.31 (16)
O3—C8—C9—C10	−10.2 (3)	C3—C2—O4—C17	−4.8 (3)
O2—C8—C9—C10	168.89 (15)		

### Hydrogen-bond geometry (Å, º)

**Table:**

*D*—H···*A*	*D*—H	H···*A*	*D*···*A*	*D*—H···*A*
C9—H9···O3^i^	0.93	2.50	3.415 (2)	168

Symmetry code: (i) −*x*+3/2, *y*+1/2, *z*.
